# Stable Iodine Distribution Among Children After the 2011 Fukushima Nuclear Disaster in Japan: An Observational Study

**DOI:** 10.1210/jc.2018-02136

**Published:** 2018-12-10

**Authors:** Yoshitaka Nishikawa, Ayako Kohno, Yoshimitsu Takahashi, Chiaki Suzuki, Hirokatsu Kinoshita, Takeo Nakayama, Masaharu Tsubokura

**Affiliations:** 1 Department of Internal Medicine, Hirata Central Hospital, Fukushima, Japan; 2 Department of Health Informatics, Kyoto University School of Public Health, Kyoto, Japan; 3 Department of Thyroid Surgery, Hirata Central Hospital, Fukushima, Japan; 4 Department of Otolaryngology and Head and Neck Surgery, Graduate School of Medicine, Kyoto University, Kyoto, Japan; 5 The Institute for Humanistic Studies, Kamakura Women’s University, Kanagawa, Japan; 6 Department of Public Health, Fukushima Medical University School of Medicine, Fukushima, Japan

## Abstract

**Context:**

Intake of stable iodine helps prevent childhood thyroid cancer in nuclear emergencies, but there is limited case information.

**Objective:**

We identified the intake rate and the factors associated with no intake among children who did not take stable iodine after the Fukushima disaster.

**Design:**

Retrospective observational study.

**Setting:**

Data were obtained from thyroid cancer screenings performed from August through November 2017.

**Participants:**

Children in Miharu Town, Fukushima, Japan.

**Intervention:**

No intervention.

**Main Outcome Measures:**

We performed multilevel logistic regression analysis at the regional and individual levels. We qualitatively examined the reasons for no intake of stable iodine based on closed- and open-ended questions.

**Results:**

The rate of distribution was 94.9%, but the intake rate was only 63.5%. Intake was lower in those aged 0 to 2 years compared with those aged ≥3 years (OR, 0.21; 95% CI, 0.11 to 0.36). Parents’ intake was positively associated with their children’s intake (OR, 61.0; 95% CI, 37.9 to 102.9). The variance partition coefficient for regions was 0.021, suggesting that the intake of stable iodine was more likely affected by individual than by regional factors. Closed-ended questions showed that the main reason for avoiding intake was concern about safety. Open-ended questions for other reasons revealed issues related to the distribution method, information about the effects and adverse effects of iodine, and instructions for iodine intake. There were no symptomatic adverse effects claimed to the town.

**Conclusions:**

The distribution and consumption of stable iodine occurred in Miharu Town after the Fukushima disaster. To prepare for future nuclear emergencies, it is important to explain to both children and parents the need for intake of stable iodine, particularly among young children.

## Teaser

The intake rate of stable iodine among children was only 63.5% in Miharu Town after the Fukushima disaster. Factors affecting the intake of stable iodine were identified.

Thyroid cancer after irradiation is a defined clinical entity, particularly among children (1). Childhood thyroid cancer after irradiation has been observed after radiation therapy or after exposure to environmental radiation after the release of radioactive iodine (1, 2). Examples of the latter include the atomic bombings of Hiroshima and Nagasaki (3), nuclear bomb testing in the Marshall Islands (4, 5), and the Chernobyl nuclear accident (6, 7). Children are at greater risk than adults of developing thyroid cancer after radiation (6), and childhood thyroid cancer after nuclear emergencies is of great public health concern.

In a nuclear emergency, intake of stable iodine is a key strategy for preventing childhood thyroid cancer along with other preventive strategies, including evacuation, sheltering, and restrictions on the consumption of contaminated food and water (8). Ingestion of stable iodine reduces internal exposure in the thyroid by blocking the uptake of radioactive iodine because it saturates the thyroid gland (8). The timing of administration is optimally between 24 hours prior to and up to 2 hours after the expected onset of exposure, but it is reasonably effective even up to 8 hours later (8). However, there is limited information about operational issues, such as the acceptability and feasibility of implementation in actual cases (8). To our knowledge, except for evidence from Poland after the Chernobyl accident (9), there is no other documented report of the intake of stable iodine as a preventive strategy in a nuclear emergency. To prepare for future nuclear emergencies, investigations of the operational issues in an actual case are needed.

**Figure 1.**
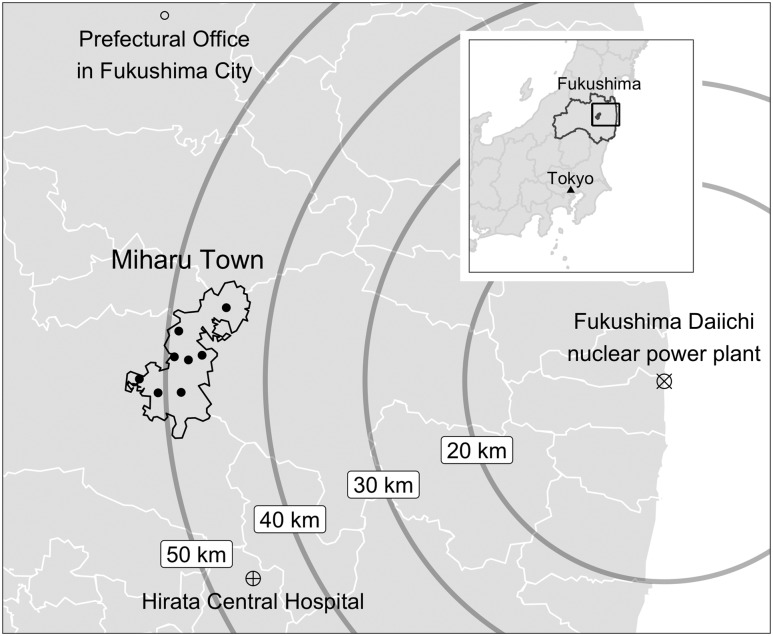
Location of Miharu Town, Fukushima Prefecture. Miharu Town, located 50 km west of the Fukushima Daiichi nuclear power plant, was the only municipality without mandatory evacuation. There were eight regions stratified by the local government, and these correspond to the eight distribution centers plotted as black dots within the town. In fiscal year 2010, a national census reported that there were 1322 children aged 0 to 9 y out of a total of 18,191 residents (22). The prefectural office, where stable iodine was stored, and Hirata Central Hospital, which conducted this thyroid screening, are also shown on the map. This map was created using R version 3.5.0 (http://www.r-project.org) and R Package “jpndistrict” (https://cran.r-project.org/web/packages/jpndistrict/index.html), which bases its geographical plotting data from National Land Numerical Information (http://nlftp.mlit.go.jp/ksj-e/index.html).

On 11 March 2011, the Great East Japan Earthquake and tsunami struck the Fukushima Daiichi nuclear power plant (F1). This resulted in the scattering of radioactive substances, including radioactive iodine, and in unintentional radiation exposure among residents (10). The amount of radioactive iodine released after the Fukushima disaster (∼520 PBq) has been estimated to be about 10% of that after the Chernobyl accident (∼5300 PBq) (11). According to the 2013 United Nations Scientific Committee on the Effects of Atomic Radiation report published after this disaster, the estimated absorbed dose to the human thyroid after the Fukushima disaster was <100 mGy (12). Therefore, the distribution of stable iodine was not required, according to World Health Organization guidelines at the time of the disaster (13). However, because of time pressure and ambiguity at the time, four local governments [those of Miharu Town (14) and three evacuated towns] distributed iodine and provided instructions for the intake of stable iodine (15). Neither the national nor the prefectural governments distributed stable iodine, even though there was sufficient stored stable iodine to do so (15). Among the towns issuing a mandatory evacuation order, it was difficult to survey all residents because the distribution status and instructions about iodine intake varied according to the place of evacuation. Miharu Town, located west of F1, was the only municipality without a mandatory evacuation order that distributed stable iodine and instructions for its intake to all children after the disaster (Fig. 1). Therefore, Miharu Town is an important case because it provides traceable information about the distribution and intake of stable iodine.

We investigated the actual distribution of stable iodine and its use by examining data from thyroid cancer screening in Miharu Town after the Fukushima disaster. This study had two objectives: (1) to identify the intake rate of stable iodine and (2) to explore the factors associated with no intake of stable iodine among those children who did not consume stable iodine. Examining the distribution and intake of stable iodine after the Fukushima disaster might be helpful in preparing for future nuclear emergencies.

## Materials and Methods

### Design, setting, and participants

This was a retrospective observational study using data from the thyroid cancer screening program performed at Hirata Central Hospital. The locations of this Hospital, Miharu Town, and F1 are shown in Fig. 1. Participants in this study were children in Miharu Town, Fukushima Prefecture, who underwent biennial thyroid screening at Hirata Central Hospital in August through November 2017. In addition to the Fukushima Health Management Survey (16), Miharu Town has continued thyroid cancer screening for all primary and secondary school students (6 to 15 years old; *i.e.*, 0 to 9 years old at the time of the disaster) in the town since 2013. To minimize recall bias, data obtained at the time of the first visit for thyroid screening before 2017 were used when available. Residents who did not live in Miharu Town before the disaster and those who did not answer the questionnaire about their intake of stable iodine were excluded from the study.

### Case of Miharu Town

Table 1 describes the time course of the Fukushima disaster and the response of Miharu Town (17, 18). On 11 March 2011, the Great East Japan Earthquake struck northeastern areas of Japan. After the establishment of a disaster countermeasure office for Miharu Town, 144 residents of the town were evacuated to public facilities.

**Table 1.** Timeline of Events and Conditions After the Fukushima Disaster and in Miharu Town

	Time	2011 Fukushima Nuclear Disaster	Time	Miharu Town
11 March	2:46 pm	Earthquake hit.		
	3:27 pm	First tsunami struck the nuclear plant (F1)	3:30 pm	Establishment of the Miharu Town Disaster Countermeasure Office
	7:03 pm	Evacuation instruction to 2 km radius from F1		Meeting of the Miharu Town Disaster Countermeasure Office
	9:23 pm	Evacuation instruction to 3 km radius from F1		
12 March	5:44 am	Evacuation instruction to 10 km radius from F1		
	3:36 pm	Explosion of Unit 1 in F1		
	6:25 pm	Evacuation instruction to 20 km radius from F1	At night	About 2000 residents of Okuma and Tomioka Town evacuated to Miharu Town
13 March			10:00 am	Meeting of the Miharu Town Disaster Countermeasure Office
				Evacuees from Tomioka Town received stable iodine
14 March	11:01 am	Explosion of Unit 3 in F1	Morning	Town officers confirmed that Prefectural Office had stored a sufficient amount of stable iodine
				Miharu Town was asked to receive stable iodine tablets for the target population (7248 residents, 3303 households)
				Public health nurses drove to the Prefectural Office to obtain tablets
			At night	Decision to distribute stable iodine (emergent meeting of the Miharu Town Disaster Countermeasure Office)
			
				Public health nurses again drove to the Prefectural Office to compensate for the shortage of stable iodine tablets
15 March	6:10 am	Explosion of Units 2 and 4 in F1	Through the night	Tablets prepared for distribution
	11:06 am	Instruction to remain indoors within a 20 to 30 km radius from F1		
		No incoming flights within a 30-km radius from F1	1:00–6:00 pm	Distribution of stable iodine with instruction of intake in each district

On 12 March, there was an explosion in the Unit 1 reactor at F1, and a mandatory evacuation order was issued for those within a 20-km radius. Miharu Town accepted about 2000 evacuees from the mandatory evacuation areas. On 13 March, evacuees from the mandatory evacuation areas received stable iodine for preventing thyroid cancer. Although there was no stockpiling of stable iodine in Miharu Town, the local government officers, in collaboration with medical doctors and pharmacists in the town, started to collect information on the effects, adverse effects and methods by which evacuees took this stable iodine.

On 14 March, there was an explosion of Unit 3 reactor at F1. The local government officers of Miharu Town confirmed that the prefectural disaster countermeasure office had sufficient stable iodine stored (400,000 tablets of potassium iodide). The prefectural office immediately approved the provision of stable iodine (potassium iodide, 50-mg tablets) for 7248 residents in Miharu Town. Public health nurses in Miharu Town collected the tablets at the prefectural office in Fukushima City, located 50 km north of Miharu Town (Fig. 1). In addition, based on the collected information about stable iodine, staff in Miharu Town created an explanatory leaflet and prepared to distribute the tablets and the leaflet to each household during that night. Accurate information on the level of radiation exposure was not obtained, but the local government decided to distribute the stable iodine tablets at night.

On 15 March, there was another explosion involving Units 2 and 4 at F1. From 1:00 to 6:00 pm, in eight distribution centers located in each region of Miharu Town (shown as eight black dots in Fig. 1), health care professionals distributed stable iodine and explained its use to residents aged under 40 years and to pregnant women. Information on the effects, possible adverse effects, target residents, contraindications for use, dose, and intake method of stable iodine for children were provided as a document. At the same time, the mayor of the town issued the recommendation of intake. There were 7248 candidates in 3303 households eligible for oral intake of stable iodine (these numbers were only for the original residents of Miharu Town). A total of 3134 households received stable iodine (94.9%).

### Data and variables

The data extracted from screening records included the age of participants at the time of the examination and at the time of the disaster; age category (infants aged 0 to 2 years and preschool/school-aged children aged ≥3 years); sex; region of residence before the disaster; whether the participant was evacuated; whether the participant (child) took stable iodine orally after the disaster; whether the parents (or people who lived with the participant) took stable iodine orally; and dietary habits, including iodine intake, as self-reported in the questionnaire. Regions of residence before the disaster were stratified into eight regions by the local government (corresponding to the eight distribution centers mentioned above and shown in Fig. 1); these are referred to as regions A to H herein.

### Questionnaire

Before visiting Hirata Central Hospital for thyroid cancer screening, all parents or guardians of the participating children were asked to complete a self-administered questionnaire at home. This included the following questions with yes or no response options: whether the participant (child) took stable iodine orally after the disaster, whether the parents or guardians took stable iodine orally, and whether the children proactively started to consume foods rich in iodine after the disaster (which might indicate health care awareness). The parents or guardians of children with no intake were asked why they did not consume potassium iodide. The following closed- and open-ended questions were asked only in 2017. Closed-ended questions regarding the reasons for no intake were included upon the advice of the local government officers and medical doctors with the following five possible multiple-choice answers: “tablets not delivered,” “concern about safety,” “no national or prefectural instruction,” “had already evacuated to other areas,” “simply forgot,” and “other reasons.” In addition to these, the survey included an open-ended question. Population perception of the degree of urgency was not obtained directly by the questionnaire.

### Quantitative analysis

Differences between the groups of children who did and did not consume iodine (intake group and no intake groups, respectively) were identified using unpaired Student *t* tests for continuous variables and *χ*^2^ tests for categorical variables. A multilevel logistic regression model was used to identify factors associated with stable iodine intake. A multilevel logistic regression model considers different levels (*e.g.*, individual and regional levels) to provide intuitive information for capturing contextual phenomena (19). There were eight regions, which was slightly less than the ideal number for multilevel regression (20); we performed multilevel logistic regression at the regional and individual levels to determine whether the effect of region outweighed the effects of other individual factors. The following individual variables were included in the model: sex, age category (infants aged 0 to 2 years and preschool/school-aged children aged ≥3 years; in this study, 3 to 9 years old), parents’ intake of stable iodine, and any increase in dietary iodine intake. All cases with complete data were used in the model. To estimate the differences between individual- and regional-level variances, the variance partition coefficient was calculated as a ratio of the regional-level error variance over the total error variance (19). The objective variable was stable iodine intake (or no intake). The regional and individual levels were evaluated to identify explanatory variables. All other variables that might have been associated with stable iodine intake were included as explanatory variables: sex, age category at the time of the disaster, whether dietary iodine intake increased, and whether the participants’ parents took potassium iodide. All statistical analyses were performed using R version 3.5.0 (http://www.r-project.org). We used the R Package “jpndistrict” for creating a map. Two-tailed *P* values <0.05 were considered significant.

### Qualitative analysis of the reasons for no intake

The questionnaire contained multiple-choice response options as mentioned above. For those who chose the “other reasons” response, the participants’ parents were given space to write comments. The open-ended comments specifying the other reasons for no intake were analyzed using thematic analysis (21) to identify the factors that affected the decision not to consume stable iodine. The authors read the written comments and assigned initial coding. A coding framework was developed by Y.N. and A.K., and Y.N. then combined the codes into categories and themes to interpret the underlying factors related to no intake. Themes were refined through discussions and were checked against the written comments by each participant and the overall dataset. Y.N. and A.K. reviewed the coding, categories, and themes and discussed these; any discrepancies were resolved through discussions. Data management and coding were performed using Excel for Mac, 2011 (Microsoft, Redmond, WA).

### Ethical considerations

This study was approved by the ethics board of Hirata Central Hospital, the Ethics Committee of the Kyoto University Graduate School of Medicine, and Fukushima Medical University Certified Review Board. Formal informed consent was obtained from all participants included in this study.

## Results

### Rate of intake of stable iodine

The total number of primary and secondary school children in Miharu Town in 2017 was 1237. Among them, 1179 children participated in the thyroid screening in 2017, of whom 966 had resided in Miharu Town at the time of the Fukushima disaster (in the fiscal year 2010, a national census reported that there were 1322 children aged 0 to 9 years in Miharu Town; see the legend for Fig. 1) (22). We excluded one child because of incomplete answers and four who were born after the Fukushima disaster. A total of 961 children were included in this study. Table 2 describes the children’s background stratified according to intake or no intake of stable iodine. The median age of the children at the time of the disaster was 5 years (range, 0 to 9 years); 63.5% (610 of 961) had taken stable iodine. There were no symptomatic adverse effects claimed to the town after the distribution of stable iodine.

**Table 2.** Children’s Characteristics

	Overall (n = 961)	Stable Iodine Intake		*P* Value
		Yes (n = 610, 63.5%)	No (n = 351, 36.5%)	
Median age at disaster, y (range)	5.0 (0–9)	5.0 (0–8)	4.0 (0–9)	<0.0001
Age category, n (%)				<0.0001
Infants (0–2 y)	174 (18.1)	85 (13.9)	89 (25.4)	
Preschool/school-aged children (3–9 y)	787 (81.9)	525 (86.1)	262 (74.6)	
Sex, n (%)				0.4096
Female	459 (47.8)	298 (48.9)	161 (45.9)	
Male	502 (52.2)	312 (51.1)	190 (54.1)	
Evacuation, n (%)				0.1132
No	583 (60.9)	383 (62.9)	200 (57.5)	
Yes	374 (39.1)	226 (37.1)	148 (42.5)	
Region, n (%)				<0.0001
A	224 (23.3)	103 (16.9)	121 (34.5)	
B	124 (12.9)	90 (14.8)	34 (9.7)	
C	221 (23.0)	144 (23.6)	77 (21.9)	
D	91 (9.5)	70 (11.5)	21 (6.0)	
E	73 (7.6)	55 (9.0)	18 (5.1)	
F	124 (12.9)	78 (12.8)	46 (13.1)	
G	75 (7.8)	49 (8.0)	26 (7.4)	
H	29 (3.0)	21 (3.4)	8 (2.3)	
Dietary iodine intake increased				0.0039
No	813 (84.6)	500 (82.0)	313 (89.2)	
Yes	148 (15.4)	110 (18.0)	38 (10.8)	
Parents’ stable iodine intake				<0.0001
No	403 (45.1)	99 (17.7)	304 (90.7)	
Yes	491 (54.9)	460 (82.3)	31 (9.3)	

Data on evacuation were missing for four participants (yes, n *=* 1; no, n = 3). Data on parents’ intake were missing for 67 participants (yes, n *=* 51; no, n = 16).

We compared the backgrounds between participants who answered the question about whether they had consumed stable iodine “yes” (n = 610) and “no” (n = 351). Age, region, increase in dietary iodine intake, and parents’ intake of potassium iodide differed significantly between the two groups (Table 2).

### Multilevel logistic regression analysis

Table 3 describes the results of the multilevel logistic regression analysis. The final model included the following explanatory variables: sex, age category at the time of the disaster, region, whether dietary iodine intake increased, and whether the participants’ parents took potassium iodide. We excluded cases where the participant was evacuated or not because the univariate analysis revealed no significant difference.

**Table 3.** Multilevel Logistic Regression Model for Intake of Stable Iodine

	OR	95% CI	*P* Value
Sex			
Female	Ref.		
Male	0.93	0.62–1.39	0.7175
Age category			
Infants (0–2 y)	0.21	0.11–0.36	<0.0001
Preschool/school-aged children (3–9 y)	Ref.		
Parents’ intake of stable iodine			
No	Ref.		
Yes	61.0	37.9–102.9	<0.0001
Dietary iodine intake increased			
No	Ref.		
Yes	1.60	0.92–2.80	0.0952

Abbreviation: Ref., reference value.

Variance partition coefficient (Region): 0.021.

Compared with children whose parents did not take stable iodine, a significantly higher percentage of children took stable iodine if their parents also did so (OR, 61.0; 95% CI, 37.9 to 102.9; *P* < 0.0001). Compared with preschool/school-aged children (3 to 9 years old), infants (0 to 2 years old) were less likely to take stable iodine (OR, 0.21; 95% CI, 0.11 to 0.36; *P* < 0.0001). The variance partition coefficient for regions was 0.021.

### Reasons for no intake of stable iodine as indicated on the multiple-choice question

The reasons for not taking potassium iodide by the 351 children who did not take iodine are shown in Table 4. Concern about safety was the most frequent reason (n = 164, 46.7%), followed by evacuation to other areas, no national or prefectural instruction, and iodine not being delivered (Table 4). Other reasons were given by 83 parents (23.6%).

**Table 4.** Reasons for No Intake of Stable Iodine After the Fukushima Disaster (N = 351)

Reason	n	%
Not delivered	27	7.7
Parental concern about safety	164	46.7
No national or prefectural instruction	34	9.7
Evacuated to other areas	36	10.3
Simply forgot	13	3.7
Other reasons	83	23.6

### Thematic analysis of other reasons for no intake of stable iodine

Among the 83 parents who gave other reasons, 75 provided descriptive comments for choosing this option. The following section describes four themes related to the decision to not take stable iodine; these were generated after careful review of the parents’ comments. Table 5 shows the coding table of the other reasons for no intake of stable iodine according to themes and categories.

**Table 5.** Coding Table for Other Reasons for No Intake of Stable Iodine

Theme	Category
Issues related to the distribution of stable iodine	No distribution
	Delayed distribution
Issues related to drug information sharing	Lack of sufficient drug information sharing on adverse effects and drug interactions
	Lack of sufficient drug information sharing about pharmacological effects
Issues related to instruction about the intake of stable iodine	Intake method for infants
	Timing of intake
Future disaster preparedness	Saving for more severe disasters
	Saving for the time of evacuation

#### 1. Issues related to the distribution of stable iodine

A few parents mentioned that they had not received stable iodine at the time of distribution.

*I had already been evacuated at the time of distribution* (P 6, a parent of a 5-year-old boy).

*Stable iodine was distributed to me on the next day in the town* (P 50, a parent of a 6-year-old girl).

#### 2. Issues related to drug information sharing

Some parents commented that they wanted more information on pharmacological effects and possible adverse effects of stable iodine.

*I could not let my child take tablets that had not been explained very well* (P 71, a parent of a 2-year-old girl).

*I was concerned about allergic reactions* (P 66, a parent of a 4-year-old girl).

#### 3. Issues related to instructions about the intake of stable iodine

In several comments, parents expressed their views that the instructions about iodine intake were difficult to understand.

*My child could not take medication because she was an infant* (P 47, a parent of a 2-month-old girl).

*I did not know the appropriate timing of the intake* (P 63, a parent of a 5-year-old boy).

#### 4. Future disaster preparedness

A few parents felt that they wanted to save the stable iodine for more severe disasters or the time of evacuation.

*I assumed that a more severe disaster would occur and saved the iodine for it* (P 4, a parent of an 8-year-old boy).

*I planned to intake at the time of evacuation* (P 5, a parent of an 8-year-old girl).

## Discussion

In Miharu Town, located 50 km west of F1, there was a high distribution rate of potassium iodide tablets to each household (94.9%), although no predistribution or stockpiling of the tablets had occurred in the village before the disaster. Despite this very high distribution rate, the intake rate was only 63.5%, even after the participants’ parents had received instructions provided by the local government. Age and parents’ intake option were factors associated with intake. Qualitative analysis revealed that concern about safety was the major reason for avoiding intake. Other issues were related to distribution methods, information about the effects and adverse effects, and instruction about intake. In future nuclear disasters, it would be important to explain to both children and parents the effects and adverse effects of iodine intake and to provide detailed instructions about the intake of iodine by infants.

Although 94.9% of households received stable iodine tablets, the intake rate was only 63.5%, which was not high despite instruction by the local government. It would be important to provide detailed information before any such disaster to both children and parents, including the intake methods for infants, timing of intake, the positive and adverse effects, and distribution methods. Our quantitative analysis showed that parents’ intake and the children’s age at the time of the disaster were associated with the children’s intake of iodine. Parents’ preferences influence children’s food preferences and decisions about treatments for children (23, 24), and it was not surprising that parents who did not consume stable iodine also did not give the tablets to their children.

The analysis of age data showed that infants were more likely not to have taken iodine compared with preschool/school-aged children. This is probably because oral medication for young children usually requires special intake procedures (25). Although a current instruction paper in Japanese has explained the use of stable iodine in a jelly formulation for young children (26), it was not available at the time of the Fukushima disaster. Although the town officers explained how to grind the tablets and mix them with drinks if necessary, it still might have been difficult for the parents to give their infants stable iodine. In some countries, national and local campaigns through local media, leaflets, and lectures have been used to increase public awareness and communication (27, 28). It would be important to provide personalized explanations to both children and parents, particularly for infants, before such a disaster.

Emergency drills, including scenarios that simulate future disasters, may help in the preparation for nuclear emergencies. In this study, compared with the mandatory evacuation order by the central government, which was nearly 100% (29), the intake rate of stable iodine was low. In general, at the time of the disaster, decisions were not made in the usual strategic manner because of the time pressure and ambiguity (30). In emergency situations, people must make quick and effective decisions under pressure based on their experience because they do not have enough time or information to make decisions analytically (31). In this study, the decision-making process about the intake of thyroid iodine was clearly divided, possibly because the Fukushima disaster forced rapid decision-making under severe pressure. As some countries conduct training for future nuclear emergencies (27), it may be important to encourage and empower residents to be prepared for nuclear emergencies by including scenarios that simulate possible future disasters.

Concern about safety was the major reason for the lack of iodine intake in this study. The issues raised by our thematic analysis should be investigated further. We found that the distribution methods, drug information about the pharmacological effects and adverse effects, and instructions about intake were associated with the decision not to take iodine. These factors probably contributed to the low intake rate of stable iodine. Issues related to the distribution method were expressed by some parents who could not obtain stable iodine at the time of distribution in the town. Periodic campaigns (*e.g.*, every 5 years) and training should be considered, as done in some other areas (27). Issues related to drug information sharing suggest that it would be important to share information about the pharmacological effects and adverse effects. In this case, no symptomatic adverse effects were claimed to the town among the residents. Workers involved in the emergency response took stable iodine continually for more than 14 days or received more than 20 tablets, and there were no immediate side effects, such as anaphylaxis or an increased rate of hypothyroidism (12). Based on the available evidence (32), information about the pharmacological effects and possible adverse effects of stable iodine should be communicated clearly. The issues related to instructions about intake indicated the importance of providing accurate instructions about the timing of intake. As reported in the guideline (8), the timing of intake is critical for an effective thyroid blockade.

Some parents saved stable iodine for future disaster preparedness. There were two categories in this theme. One was that some parents wanted to save it for more severe disasters. The other was that some parents saved stable iodine for the time of evacuation because going outside would be a risk of radiation exposure immediately after the disaster. In the closed-ended questions, some participants answered that they did not take stable iodine because they had received no national or prefectural instruction. The intake rate was low, possibly because there were residents who perceived the instruction of stable iodine intake as not requiring immediate action. Ideally, instructions about intake should be given before a disaster, as done in some countries (27). Because the decision of whether to take stable iodine would be made by each resident, the local government should clearly issue instruction orders and the timing of intake with recommended levels.

Arranging for the distribution by local government officers should involve regional managers who are acquainted with the local geographical situation. In this case, prefectures stockpiled the stable iodine tables, and the town officers drove to the prefectural office to obtain the tablets and prepare them for distribution to the target population. In some European countries, local stockpiling occurs (27) and would shorten the time needed for distribution. Implementation of a distribution system by prefectural crisis management services in cooperation with local governments may also be a good practice, as done in Poland (9, 27). In this case, the local government and each regional manager organized all eight distribution centers. The residents were familiar with the regional managers and the distribution centers (publicly used community centers and health care centers). Regional managers notified all residents in their corresponding regions and distributed stable iodine individually if a resident could not obtain stable iodine at the time of distribution. This distribution method played an important role in the high distribution rate. The lower intake rate or intake compared with the distribution rate is less likely to have been affected by a regional factor (place of distribution) compared with the individual factors noted above. It would be helpful to provide a personalized explanation for each resident (household) by regional managers at the time of distribution.

This study had some limitations. First, the thyroid screening questionnaire was conducted in only one town, and the data obtained might not be representative of all areas of Fukushima Prefecture. Second, this study was performed using data obtained in 2017, 6 years after the disaster, and there might have been recall bias. However, to avoid recall bias as much as possible, data obtained at the time of the first visit for thyroid screening before 2017 were used if these were available. Third, public perception of the degree of urgency was not directly obtained by the questionnaire, so differences in urgency perception would affect their decision about stable iodine intake. Because there was not enough information on the expected radiation levels in Miharu Town (14), there would have been some residents who perceived that it was not urgent to take stable iodine, as revealed in our qualitative analysis. Fourth, background data of the parents, such as age, sex, and educational status, were not collected because this study was performed using the thyroid screening data obtained by town officials. Although we used the data on parents’ intake of stable iodine, we could not know the parents’ age or whether the parents were targets of iodine distribution. However, many of the parents in this study would have been targets of iodine distribution (<40 years old), given that the median age of the children at the time of the disaster was 5 years. Our findings may be useful in preparation for future nuclear emergencies.
